# Biocontrol of Fruit Rot of *Litchi chinensis* Using Zinc Oxide Nanoparticles Synthesized in *Azadirachta indica*

**DOI:** 10.3390/mi13091461

**Published:** 2022-09-03

**Authors:** Junaid Ahmed, Musrat Ali, Huda M. Sheikh, Manal O. Al-Kattan, Farhana, Urooj Haroon, Masoumeh Safaeishakib, Mahnoor Akbar, Asif Kamal, Mohammad Sameer Zubair, Muhammad Farooq Hussain Munis

**Affiliations:** 1 Department of Plant Sciences, Faculty of Biological Sciences, Quaid-i-Azam University, Islamabad 45320, Pakistan; 2 Department of Biology, College of Science, University of Jeddah, Jeddah 21589, Saudi Arabia; 3 Department of Plant Pathology, University of California, Davis, CA 91616, USA; 4 Department of Biology, Science and Research Branch, Islamic Azad University, Tehran 1477893855, Iran

**Keywords:** fruit rot, lychee, ZnO NPs, *Azadirachta indica*, *Aspergillus niger*

## Abstract

Lychee (*Litchi chinensis* Sonn.) is a famous fruit species of tropical and subtropical regions of the world and many biotic and abiotic stresses affect its yield. In this study, lychee fruit rot has been observed and its incidence has been controlled by using zinc oxide nanoparticles (ZnO NPs). Diseased lychee fruits were collected and diagnosed to identify disease-causing pathogens. Morphological appearance, microscopic observation, and sequence analysis of the amplified ITS region identified this isolated pathogen as *Aspergillus niger*. To control this problem, ZnO NPs were prepared in the leaf extract of *Azadirachta indica*. Before their antifungal activity, ZnO NPs were characterized using sophisticated approaches. FTIR revealed the presence of reducing and stabilizing molecules on ZnO NPs including alcohol, carboxylic acid, alkyl halide, amine, and alkyl halide. Crystalline nature and average size (29.024 nm) of synthesized ZnO NPs were described by X-ray diffraction. EDX analysis depicted the mass percentage of zinc (30.15%) and oxygen (14.90%). SEM analysis displayed the irregular shape of nanoparticles and confirmed the nano-size of ZnO NPs. Maximum mycelial growth inhibition (70.5%) was observed at 1.0 mg/mL concentration of ZnO NPs in vitro. In in-vivo disease-control analysis, maximum control of lychee fruit rot disease was observed at the same concentration. These results reveal the potential use of these ZnO NPs on a larger scale to replace hazardous chemical fungicides.

## 1. Introduction

Lychee (*Litchi chinensis* Sonn.), also spelled *litchi* or lichi, is cultivated in tropical and subtropical regions of the world. It belongs to the *Sapindaceae* family of soapberries. Lychee has a rough and thin pericarp and after ripening, it becomes soft, red, and cracked (due to the presence of brecky sclereids in the fruit that prevent them from any kind of physiological stress or mechanical damage [1]). This plant has been reported to have originated from South China, Northern Vietnam, and India [2,3]. In the 18th century, the lychee plant was introduced to India through Burma, and after that, it spread into many countries of the world. Lychee is a famous fruit in the sub-continent, and it is an emerging crop in Pakistan, where it is considered as the queen of fruits. In Pakistan, lychee is cultivated on 572 ha, and annually, 9250 tons of lychee are produced. Due to less production and high disease infestation, its demand is high, and it is sold at a good price in the markets [4].

Lychee fruit is affected by various living and non-living environmental factors. Among all, fungal diseases are the most common to cause pre-harvest and post-harvest losses. Other pathogens such as bacteria and viruses also cause fruit diseases of lychee. Anthracnose is the most frequent disease and it has been reported to be caused by *Colletotrichum gloeosporioides* Penz. Fruit rot has also been described to be caused by an oomycete (*Peronophythora litchi*). It mostly attacks leaves, panicles, and fruits. Due to their attack, a brownish color is produced on immature fruit, and sometimes white-colored mildew also appears on the fruit skin. Fruit blight is another well-documented post-harvest disease of lychee. *Alternaria alternata* is the causal agent of this disease [5].

To overcome these disease-causing agents, different kinds of chemical pesticides have been used for many decades. Chemical pesticides including chlorpyrifos, deltamethrin, and imidacloprid have been used on different parts of lychee trees [6]. These pesticides are very toxic and after a few years, pathogens also develop a defense mechanism against these types of chemicals. Therefore, scientists are focusing on discovering environmental and health-friendly technologies to control diseases of fruits and other edible parts [7]. In comparison with chemical fungicides, bio-degradable natural fungicides do not harm crops, soil, or the environment [8]. Nanotechnology is one of the emerging fields to use natural substances for the control of plant diseases. The preparation of nanoparticles in plants and other biotic agents gives them antibiotic properties [9]. These nanoparticles are health-friendly and exhibit great potential for disease control. Disease-causing pathogens also show no resistance or adaptability to these nano bio-fungicides [10]. Different metabolites, present in plant extracts, act as capping and reducing agents and provide antibiotic properties to synthesized nanoparticles [11]. The nanoparticles synthesized in plant extracts are also called “green nanoparticles” and these nanoparticles have been used to control a variety of plant diseases. The synthesis of these nanoparticles is cost-effective and eco-friendly [12].

This study has been designed to optimize the synthesis of green ZnO NPs in the leaf extract of *Azadirachta indica.* Characterization of synthesized ZnO NPs was performed by using XRD, SEM, UV-visible spectroscopy, FTIR and EDX. Successfully synthesized green ZnO NPs were used to control the lychee fruit disease.

## 2. Materials and Methods

### 2.1. Collection of Rotten Lychee Fruits

Lychee fruit with fruit-rot symptoms were collected from National Agricultural Research Centre (NARC), Islamabad (33.6701° N, 73.1261° E). The collected samples of diseased fruit were placed in individual polythene bags and transported to the laboratory for further analysis.

### 2.2. Isolation of Disease-Causing Pathogen

Sterilization of diseased lychee fruit was performed with 70% ethanol and air-dried. Using a sterile scalpel, diseased fruit parts were excised (roughly 3 mm in diameter) and aseptically placed on Petri plates containing PDA media and incubated for 5–6 days at 25 ± 2 °C. To obtain a pure culture of the disease-causing pathogen, the emerging fungal colonies were constantly sub-cultured.

### 2.3. Morphological and Microscopic Identification of Pathogen

The purified cultures of the isolated pathogen were observed after one week. Several cultural and morphological characteristics including colony color, colony development pattern, and conidial morphology were noted. For the microscopic study of grown mycelium, the standard methodology was followed [13]. For this purpose, a drop of lactic acid was placed on a clean glass slide. With the help of a sterilized inoculating needle, a tiny part of the aerial mycelia from the pure culture was collected and placed on the slide. Then a drop of lactophenol blue was added and a coverslip was placed carefully to avoid air bubbles. The slides were blot-dried and observed under a light microscope at 100× magnification to examine spores, hyphae, and other pathogenic characteristics.

### 2.4. Molecular Identification of Fungal Species

One-week-old fungal colonies were used for the extraction of DNA, using the CTAB method [14]. The ribosomal internal transcribed spacer region (ITS) was amplified using forward (ITS1) and reverse primers (ITS4). Sequencing of the amplified PCR product was conducted and the similarity of the resultant sequence was determined by BLAST analysis in NCBI GenBank. MEGA-7 software was used to construct a phylogenetic tree to obtain the evolutionary relationship of obtained sequence with associated sequences.

### 2.5. Pathogenicity Test

Pathogenicity of disease-causing fungus was investigated. For this purpose, healthy, fresh, mature, and unripe lychee fruit were sterilized with 70% ethanol and washed with distilled water. Using a cork borer, holes of 3-mm size were produced, and mycelial discs of 7-day-old fungal cultures were placed in these holes. Each hole was thoroughly sealed with petroleum jelly. The control was inoculated with distilled water. All fruit were covered with autoclaved muslin cloth and placed in an incubator at 25 ± 2 °C. After 5 to 10 days of the inoculation, disease symptoms were observed.

### 2.6. Preparation of Leaf Extract of Azadirachta indica

Leaves of *A. indica* were collected from Rahim Yar Khan. Collected plant material was washed properly to clean dust particles and shade-dried to reduce moisture. After 10–15 days, dried leaves were ground into a fine powder, mixed in 500 mL distilled water, and boiled for 30 min. The mixture was cooled down and filtered through muslin cloth and Whatman filter paper no. 1. The clear filtrate was stored at 4 °C until further use.

### 2.7. Preparation of ZnO Nanoparticles

For the preparation of ZnO nanoparticles (ZnO NPs), 1 mM salt of zinc chloride was mixed with the extract of *A. indica* in a 1:2 ratio. Using a hot plate, the mixture was stirred for 2 h at 70 °C. Thereafter, it was calcinated to powder form and characterized further.

### 2.8. Characterization of Nanoparticles

Before antifungal activity analyses, synthesized ZnO NPs were characterized by using the following sophisticated techniques.

#### 2.8.1. UV-Visible Spectroscopy

ZnO NPs were characterized by measuring the ultraviolet-visible absorbance spectrum of the solution in distilled water, using a UV-vis spectrophotometer (V-670 UV-VIS, JASCO). NPs were scanned for spectrophotometric analysis, keeping the distilled water as a blank [15]. The scanning range was maintained between 400–700 nm, using a double beam at 1 nm resolution. 

#### 2.8.2. Fourier Transformed Infrared (FTIR) Spectroscopy

By using the KBr pellet method, FTIR spectroscopy was performed to detect and analyze different functional groups present on the surface of ZnO NPs. The prepared NPs were compressed with 150 mg KBr and evaluated in the spectrum ranges of 4000 cm^−1^ to 400 cm^−1^ by employing a Bruker, Vertex 70, FTIR spectrometer.

#### 2.8.3. XRD Analysis

The structure and size of ZnO NPs were determined using XRD analysis. X’Pert High Score software was used to detect crystallographic characteristics while the size of prepared ZnO NPs was calculated by the following equation:

D = kλ/βcosθ(1)

where D shows the crystalline size perpendicular to the reflecting planes; K denotes the shape factor; λ indicates X-ray wavelength; β reflects the full width at the half maximum (FWHM) and θ is the diffraction angle.

#### 2.8.4. Scanning Electron Microscopy (SEM) and Energy Dispersive X-ray (EDX) Analysis

Sonication of prepared ZnO NPs was performed for 5 min in double-distilled water to make their solution. On double-carbon-coating conductive tape, a drop of solution was placed and dried under the lamp. For SEM and EDX analysis, an ionic emission SEM system (VEGA3 TESCAN) was used.

### 2.9. Mycelial Growth Inhibition Assay, In Vitro

For the estimation of the mycelial growth inhibition ability of synthesized ZnO NPs, the poisoned food technique was used. Prepared ZnO NPs were mixed in PDA media and the media were poured on Petri plates with five different concentrations (1.0 mg/mL, 0.75 mg/mL, 0.5 mg/mL, 0.25 mg/mL, and 0.1 mg/mL). In the middle of each Petri plate, 5 mm of fungal inoculum was placed. PDA Petri plates with no NPs served as control. Three plates for each treatment were inoculated with the fungus and incubated at 25 °C. The diameter of the fungal colony was determined after 7 days of incubation, and mycelial inhibition was determined by using the following formula:

Growth inhibition % = C − T **÷** C × 100 (2)

where C represents the average mycelial growth in control and T is the average mycelial growth in treated Petri plates.

### 2.10. Disease Control Assay, In Vivo

To see the efficiency of ZnO NPs in controlling lychee fruit rot, 15 fresh lychee fruits were inoculated with the identified fungal spore concentration (1 × 10^6^ spores/mL). After 2 days of inoculation, three fruit were sprayed (until run-off) with each of the selected four concentrations of ZnO NPs (1.0 mg/mL, 0.75 mg/mL, 0.5 mg/mL, 0.25 mg/mL and 0.1 mg/mL). The remaining three control fruits were sprayed with distilled water. Inoculated and treated fruit with NPs were covered with autoclaved muslin cloth and retained in an incubator at 25 ± 2 °C. After 7 days of the inoculation, disease symptoms were observed.

### 2.11. Statistical Analysis

The experiment was carried out in three replicates. Mean and standard deviation were obtained using MS-Excel 2016 software (Microsoft Inc., Albuquerque, NM, USA).

## 3. Results

### 3.1. Morphological and Microscopic Identification of the Pathogen

**Figure 1:**
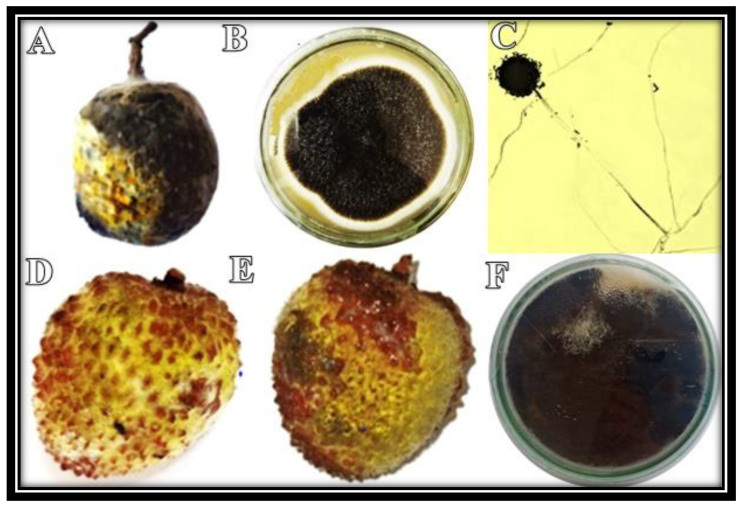
Fruit rot of lychee was observed in the field (**A**). Disease-causing pathogen was isolated on PDA (**B**). Microscopic examination of fungus was carried out at 100× magnification (**C**). The fungus was re-inoculated, and symptoms were observed after 5 days (**D**) and 10 days of inoculation (**E**). The pathogen was re-isolated on PDA (**F**).

Rotten lychee fruit was collected (Figure 1A) and the pathogen was successfully isolated on PDA media. The isolated pathogen was blackish from the center and its edges appeared whitish grey (Figure 1B). The microscopic photograph revealed black radial conidial heads. The conidiophores were hyaline and smooth, and they were darkening towards the vesicle. Conidial heads were dark brown to black, globose to sub-globose, and had a rough wall (Figure 1C).

### 3.2. Pathogenicity Test

Large water-soaked lesions appeared on the surface of the lychee fruit after 5 days of inoculation (Figure 1D). After 10 days of inoculation, light to dark, water-soaked lesions were observed on the surface of the fruit, which gradually grew and coalesced, resulting in fruit rot (Figure 1E). The growth pattern of the re-isolated fungus was similar to the initially isolated fungus (Figure 1F).

### 3.3. Molecular Identification and Phylogenetic Analysis

**Figure 2:**
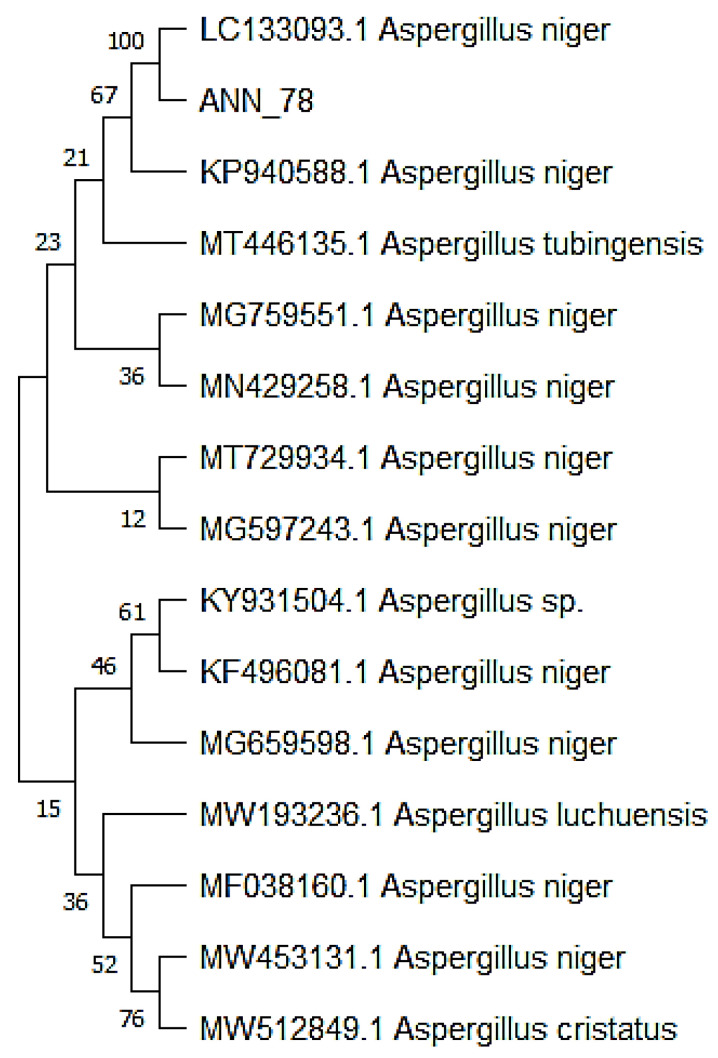
Phylogenetic analysis of obtained sequence with 14 related sequences.

Amplified rDNA sequence of isolated pathogen showed 100% similarity with *Aspergillus niger* (LC133093.1). Phylogenetic analysis revealed the evolutionary relationship of our fungus with *A. niger* (LC133093.1) by placing them in the same clade (Figure 2).

### 3.4. UV-Visible Spectroscopy

**Figure 3:**
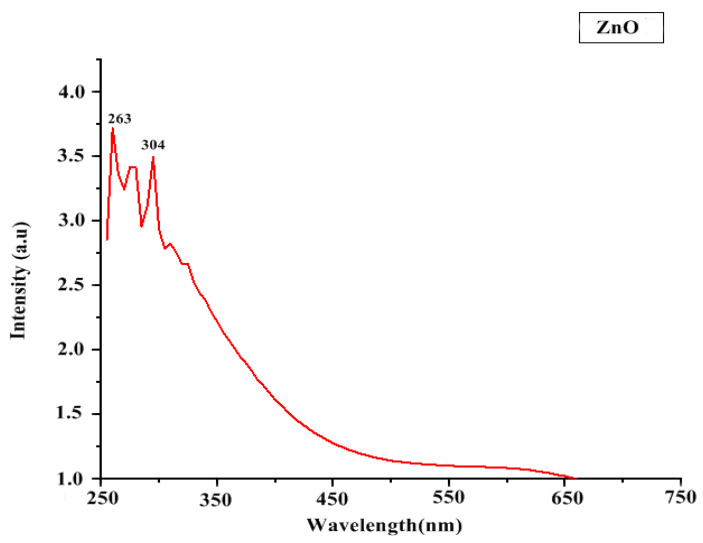
UV Spectroscopy of ZnO NPs.

In UV-Vis spectroscopy, the maximum absorbance of ZnO NP solution was found in the range of 250–350 nm (Figure 3). It indicates the presence of highly effective organic molecules (ZnO NPs) in the solution.

### 3.5. FTIR Analysis

**Figure 4:**
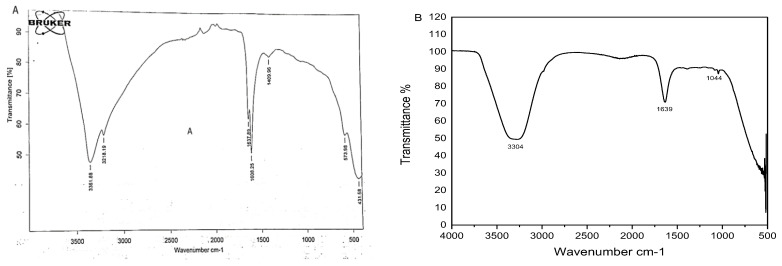
(**A**) FTIR spectrum of green ZnO NPs (**B**) FTIR spectrum of leaf extract of *Azadirachta indica*.

FTIR spectra revealed a prominent peak in the range of 500 to 5000 cm^−1^, indicating a metal-oxygen bond on ZnO NPs (Figure 4A). The absorption band at 3361.68 cm^−1^ represented O-H stretching of water molecules, broad peak at 3218.19 cm^−1^ indicated O-H stretching of carboxylic acid, two different peaks at 1637.89 cm^−1^ (C=C stretching) and 1608.25 cm^−1^ (N-H bending) suggested the presence of amine groups, a peak at 1409.96 cm^−1^ represented O-H alcohol bending and the peak at 573.98 cm^−1^ indicated the presence of alkyl halides (Table 1). Alcoholic and alkyl halide peaks were not present in the FTIR spectra of simple plant extract (Figure 4B, Table 2).

### 3.6. XRD Analysis

**Figure 5:**
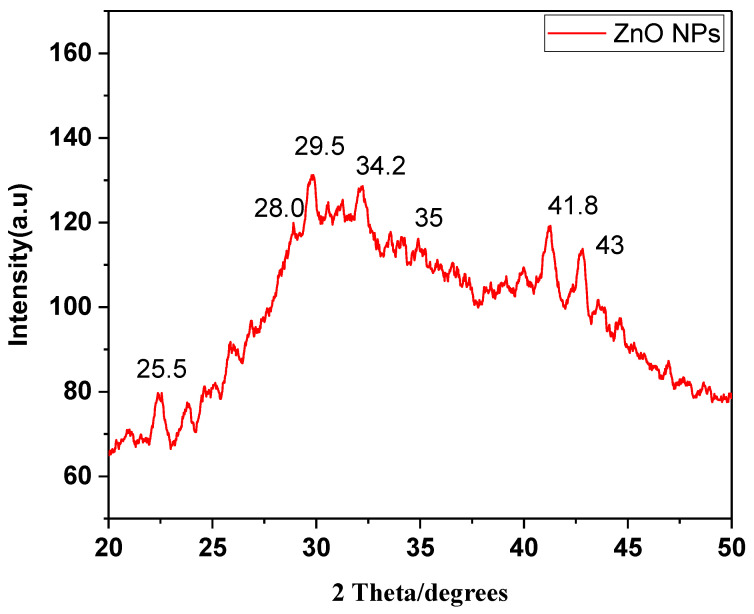
XRD analysis of ZnO NPs synthesized in *A. indica*.

XRD analysis was successfully performed at a scanning rate of 1° per minute in 0.013° increments, encompassing the 2-theta angle between 10° and 80°. The pattern revealed five diffraction and distinctive peaks of ZnO NPs (Figure 5). XRD spectra revealed prominent peaks at 25.5, 28.0, 29.5, 34.2, 35, and 41.8°, corresponding to the morphology of ZnO NPs. The planes of the XRD patterns correlate well with JCPDS number 1314-13-2. The average size of ZnO NPs was observed as 29.024 nm.

The crisp and powerful peaks clearly demonstrated the crystalline nature of ZnO NPs.

### 3.7. SEM and EDX Analysis

**Figure 6:**
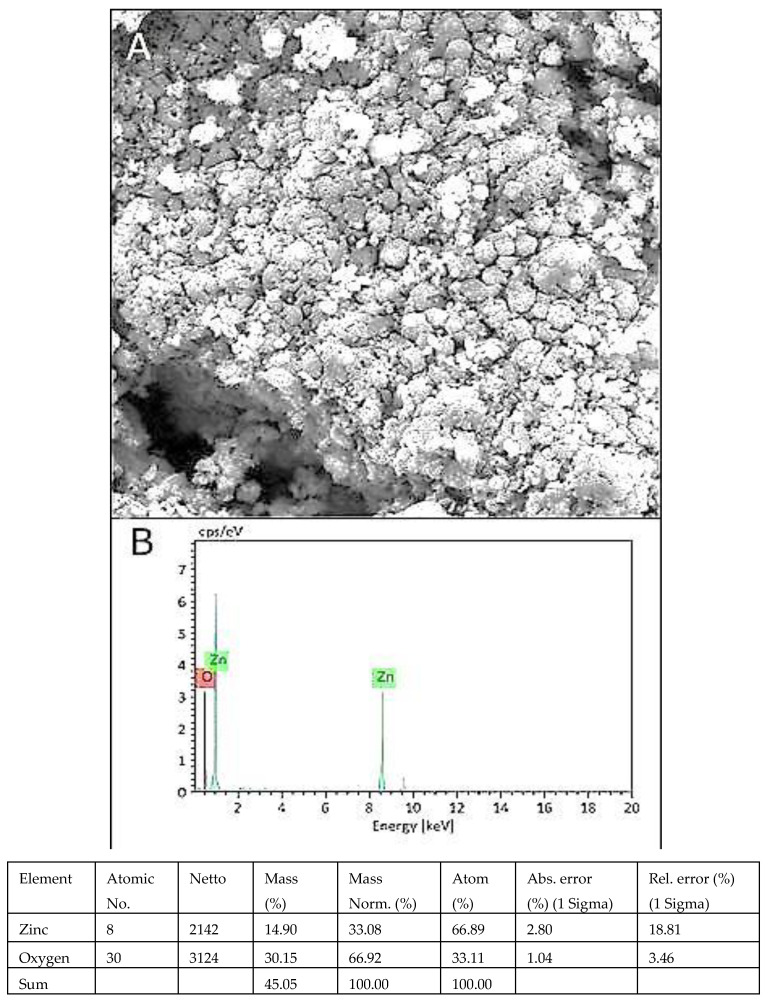
SEM image of ZnO NPs (**A**), EDX spectrum of ZnO NPs (**B**).

The morphology of green ZnO NPs was efficiently evaluated by SEM examination (Figure 6A). Prepared ZnO nanoparticles were irregular in shape. The EDX spectrum described the elemental makeup of ZnO NPs and showed strong peaks of Zn and O (Figure 6B). Mass percentage of oxygen and zinc was recorded to be 30.15% and 14.90%, respectively. Peaks of zinc and oxygen confirmed the synthesis of ZnO NPs in the studied material.

### 3.8. Antifungal Activity of ZnO NPs, In Vitro

**Figure 7:**
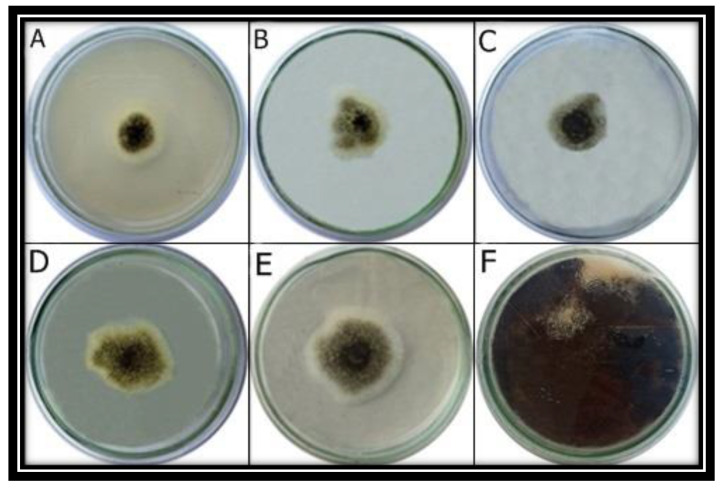
Antifungal activity analysis of ZnO NPs, in vitro. *A. niger* was grown at different concentrations of ZnO NPs including 1 mg/mL (**A**), 0.75 mg/mL (**B**), 0.5 mg/mL (**C**), 0.25 mg/mL (**D**) and 0.1 mg/mL (**E**). ZnO NPs were not supplemented in control (**F**).

The growth inhibition of the pathogen was seen at all concentrations of NPs (Figure 7). The greatest growth inhibition under in vitro conditions was observed at 1.0 mg/mL concentration of ZnO NPs (Table 3).

### 3.9. Antifungal Activity of ZnO NPs, In Vivo

**Figure 8:**
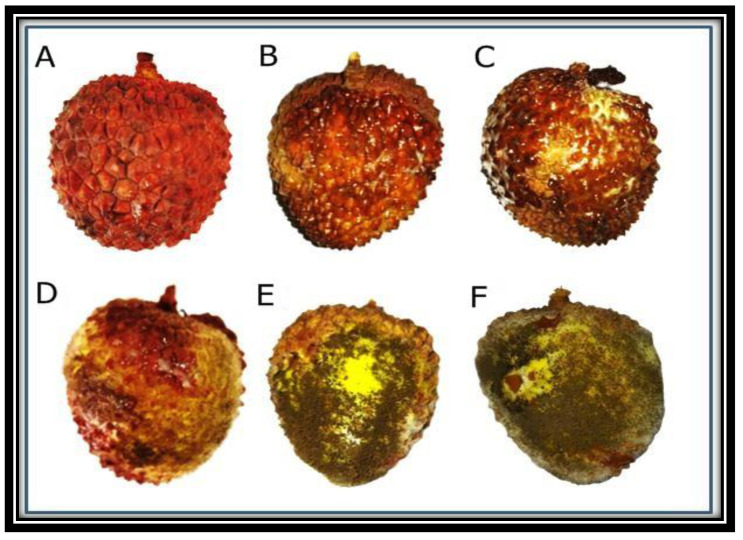
Lychee fruit were infected and treated with different concentrations of ZnO NPs including 1 mg/mL (**A**), 0.75 mg/mL (**B**), 0.5 mg/mL (**C**), 0.25 mg/mL (**D**) and 0.1 mg/mL (**E**). Control plants were not treated with any NPs (**F**).

Different concentrations of ZnO NPs displayed variable control of fruit rot disease of lychee (Figure 8). The disease of the fruit was controlled by different concentrations of the NPs but the maximum reduction in the disease incidence was observed at 1.0 mg/mL concentration of ZnO NPs (Table 4). In this in-vivo analysis, the maximum effectiveness of ZnO NPs was also observed at 1.0 mg/mL concentration (Figure 8).

## 4. Discussion

Fungi are considered as the most destructive plant pathogens [16]. Spoilage caused by fungi leads to the alteration in food quality and changes its taste, odor, and appearance. In the present study, *Aspergillus niger* was identified as the fruit rot pathogen of lychee. *A. niger* has previously been reported to cause fruit rot of grapefruit and lemon in Pakistan [14]. In few other studies, *A. niger* has been documented for its involvement in both pre-harvest and post-harvest losses of citrus, mango, and pomegranate [17,18].

In this study, to control lychee fruit disease, biodegradable, eco-friendly, and low-cost ZnO NPs were applied. These ZnO NPs were prepared in the plant extract of *Azadirachta indica.* Previous research reports have described the antifungal potential of *A. indica* [19]. According to published reports, many pathogenic fungi including *Botrytis ceneria*, *Magnaporthe* spp., *Collettrichum* spp., *Pythium* spp. and *Aspergillus flavus* have been controlled by green ZnO NPs [20]. Results of UV-Vis spectroscopy of this study were in agreement with previous results [9]. The size of green synthesized ZnO NPs was small, with a large surface area. The diffraction pattern of the XRD showed the highly crystalline nature of ZnO NPs. Antimicrobial properties of NPs are highly influenced by their crystalline structure and size [21].

The FTIR spectrum successfully identified functional groups. These are bioactive molecules and act as capping and stabilization agents on NPs [22]. In the FTIR spectra, the presence of amine groups revealed the existence of proteins around ZnO NPs. These results confirmed that the protein molecules of the extract function as reducing and stabilizing agents by attaching to ZnO NPs, with the help of a free primary amino group [23]. These peaks also confirmed the presence of primary alcohol and other bioactive compounds that are secreted by *A. indica*. The presence of aromatic alcohols and alkyl halides compounds on the surface of NPs may explore their potential in imparting antifungal properties on these NPs [24]. SEM was performed for the morphological analysis of NPs. Our green synthesized ZnO NPs were irregular in shape, which is consistent with previous results [25]. The EDX spectrum indicated the highest optical absorption peaks of Zn and O, which confirms the successful production of ZnO NPs [15].

The effective role of ZnO NPs in fungal growth inhibition was observed at 1.0 mg/mL concentration. NPs are very efficient in rupturing cell membranes, suppressing cell division, and inhibiting the formation of the cell wall. NPs break the cell membrane by blocking ergosterol production, interacting with sterol, and producing pits, resulting in membrane leakage and cell death [26].

## 5. Conclusions

This study has described the involvement of *A. niger* in causing lychee fruit rot. This is first report of this disease in Pakistan. The leaf extract of *A. indica* contains bioactive components that efficiently perform capping and stabilization of ZnO NPs. These small-size NPs can considerably suppress the development of lychee fruit disease at 1.0 mg/mL concentration of ZnO NPs. For a sustainable environment, these bio-pesticides or green synthetic NPs should be used in the field to replace hazardous chemical pesticides.

## Figures and Tables

**Table 1:** FTIR spectra of ZnO NPs synthesized in *A. indicae*.

S. No.	Functional Groups	Compound	Wave Number	Type of Vibration	Bonding	Peaks
1	OH	Alcohol	3361.68	Stretching	Strong	Broad
2	O-H	Carboxylic Acid	3218.19	Stretching	Strong	Broad
3	-C=C	Conjugated Alkene	1637.89	Stretching	Medium	Broad
4	N-H	1° Amine	1608.25	Bending	Medium	Broad
5	S=O	Sulfate	1409.96	Stretching	Strong	Broad
6	C-Br	Alkyl Halides	573.98	Stretching	Strong	Broad

**Table 2:** FTIR spectra of ZnO NPs synthesized in *A. indicae*.

S. No.	Functional Groups	Compound	Wave Number	Type of Vibration	Bonding	Peaks
1	O-H	Alcoholq	3304.21	Stretching	Strong	Broad
2	-C=C	Alkene	1639.16	Stretching	Medium	Broad
3	N-H	Anhydride	1044.48	Bending	Medium	Broad

**Table 3:** Growth inhibition of *A. niger* at different concentrations of ZnO nanoparticles.

Concentration (mg/mL)	Percentage Inhibition (%)
1.0	70.51 ± 3.41
0.75	60.11 ± 3.31
0.50	43.18 ± 3.73
0.25	29.45 ± 2.11
0.1	21.92 ± 1.98
Control	0.00

**Table 4:** After treatment of different concentrations of ZnO NPs on diseased lychee fruit, the disease area is shown here.

Treatment	Diseased Area (mm)
1.0	25.4 ± 2.4
0.75	33.2 ± 3.2
0.50	40.6 ± 5.0
0.25	50.7 ± 4.8
0.1	89.6 ± 2.8
Control	92.0 ± 2.1

## Data Availability

Not applicable.
